# Social power motives, gendered traits, and aggression in eSports: evidence from two survey waves

**DOI:** 10.3389/fpsyg.2026.1833661

**Published:** 2026-05-13

**Authors:** Wenrong Cui

**Affiliations:** Department of Communication Studies, The University of Kansas, Lawrence, KS, United States

**Keywords:** dominance-prestige-leadership motives, eSports, honor of kings, in-game aggression, masculinity–femininity traits

## Abstract

**Introduction:**

This study examined how social power motives and gendered traits relate to in-game aggression among Honor of Kings players.

**Methods:**

Two survey waves were conducted with HoK players: Wave 1 included 295 participants, and Wave 2 included 194 returning participants. Participants completed measures of dominance, prestige, leadership, masculinity, femininity, and in-game aggression.

**Results:**

Dominance consistently related to higher in-game aggression. Prestige also showed positive associations with aggression. Leadership showed negative associations only when modeled with dominance, so this finding should be interpreted cautiously. Femininity consistently related to lower aggression, whereas masculinity did not independently relate to aggression.

**Discussion:**

Aggression in this eSports context is most consistently linked to dominance-oriented status striving. Femininity may protect against aggressive in-game behavior.

## Introduction

Competitive digital games such as online multiplayer games grouped under “eSports” continue to expand globally, evident in the steady growth of both participants and audiences (Borggrefe and Hoffmann, 2024). Online multiplayer environments, including mobile gaming platforms, also operate as expansive competitive arenas where community interaction, status pursuit, and aggression intersect (Britt and Britt, 2020). Toxic behavior remains pervasive across these mobile gaming spaces and imposes documented psychological and community burdens (Zsila et al., 2022). Global participation continues to expand, about 3.2 billion players in 2024, including more than 742 million in China (Gan et al., 2024; Katatikarn, 2024). However, research has focused more on prevalence than on the psychological mechanisms that produce aggression. Even though competitive video games are becoming huge, researchers still do not use them enough to study human behavior (Borggrefe and Hoffmann, 2024; Mora-Cantallops and Sicilia, 2018). The field still lacks a sufficiently clear account of how status-related motives and gendered traits are associated with aggression in competitive gaming contexts.

A first issue concerns power motives. Power motives (dominance, prestige, and leadership) represent distinct strategies for achieving social status within the DPL framework (Suessenbach et al., 2019). More foundational work on status hierarchies conceptualizes dominance and prestige as two distinct routes to social rank, with dominance relying on coercion and prestige on freely conferred respect for competence (Henrich and Gil-White, 2001; Cheng et al., 2013). Consistent with this distinction, dominance has been linked more directly to aggression and intimidation than prestige (Johnson et al., 2007). Leadership reflects a related but distinct orientation toward social influence within the DPL account (Li et al., 2024b).

A second issue concerns gendered personality traits. Masculinity and femininity, treated as independent dispositional dimensions rather than proxies for biological sex (Bem, 1981), may strongly relate to the social power motives and predict aggression patterns. Masculine traits align with assertiveness and confrontational behavior in gaming contexts (Wright, 2020), whereas feminine traits correspond to restraint and lower aggression (Liu et al., 2024). Recent work shows that femininity and androgyny can weaken the link between status-oriented dispositions, moral disengagement, and cyberaggression (Gan et al., 2024). In mobile gaming/eSports cultures that valorize toughness and competitive display, these gendered traits likely shape how players pursue status and respond to conflict (Cote, 2015; Fox and Tang, 2016).

In-game aggression, including verbal hostility, targeted harassment, and other behaviors intended to harm, reflects the intersection of these motives and traits (Bryant and Smith, 2001). Competitive pressure and online disinhibition intensify cyberaggression (Gan et al., 2024), making online games an especially suitable context for testing how motivational and personality factors drive hostile behavior.

Overall, this study addresses these gaps by examining how distinct power-motive profiles and gender-trait orientations relate to in-game aggression. The goal is to move beyond documenting in-game aggression, instead clarifying how these psychological characteristics are associated with aggressive conduct within competitive gaming environments. To strengthen the design, the study used two survey waves with the same participant pool across an interval. The purpose of the second wave was to examine whether the main association patterns among social power motives, gendered traits, and in-game aggression would reappear over repeated measurement. Accordingly, the two-wave design is used here to assess pattern recurrence and short-interval consistency. Beyond the specific case of HoK, the study also speaks to a broader question in digitally mediated interaction: how status striving, and gendered self-concepts are associated with aggressive behavior in environments structured by competition, partial anonymity, and rapid social evaluation. In this sense, the present study is relevant not only to eSports research, but also to wider efforts to understand conflict, reputation management, and prosocial versus coercive influence in virtual social settings.

## Literature review

### Honor of kings

*Honor of Kings (HoK)* is a multiplayer online battle arena (MOBA) on mobile phones developed by TiMi Studio and published by Tencent Games. Since its 2015 release, it has become the dominant MOBA title in mainland China (Yao and Chen, 2022) and has achieved substantial global reach (ActivePlayer, 2025). The core mechanics involve two teams of five players competing to destroy the opposing base, with each player selecting a hero whose role shapes strategic options (Liu and Agur, 2023). During standard gameplay, players coordinate primarily through in-game text and preset communication systems rather than face-to-face interaction, and they do not normally see one another directly. In addition, players are not necessarily aware of other players’ offline identities or gender unless such information is disclosed through usernames, profile cues, or voluntary communication. HoK also supports a large competitive ecosystem, including the Honor of Kings Professional League (KPL), established in 2016 with two annual seasons (Liu, 2024). This structure creates a stable context for observing status dynamics, coordinated action, and conflict behaviors in the mobile gaming area.

### Dominance, prestige, and leadership interconnections in online games

Contemporary hierarchy research identifies three separable social power motives (i.e., dominance, prestige, and leadership) that capture distinct strategies for attaining influence (Suessenbach et al., 2019). Dominance reflects coercive control; prestige reflects influence gained through competence and earned respect; leadership entails responsibility-taking and initiative for collective goals (Moynihan et al., 2023; Suessenbach et al., 2019). These motives share a higher-order drive for influence yet diverge in emotional correlates and behavioral tendencies (Marion-Jetten and Schattke, 2022). Dominance aligns with anger and aggression, prestige with reputation management, and leadership with emotional stability and prosocial behavior (Suessenbach et al., 2019). Evolutionary dual-strategy theories position dominance and prestige as distinct routes to rank, with prestige eliciting admiration and dominance eliciting distrust (Henrich and Gil-White, 2001; Cheng et al., 2013).

Online games function as micro-societies in which these strategies become observable in concrete behaviors (Molyneux et al., 2015; Warmelink and Siitonen, 2011), including team-based struggles over territorial control in mobile titles such as *Pokémon Go* (Woods, 2020). High-dominance players may impose authority through harsh coordination, coercive communication, or aggressive displays such as exploiting early-game advantages to constrain teammates and opponents. Prestige-oriented players acquire influence through skill demonstration, mastery of game mechanics, leaderboard achievement, or mentoring behavior (Kusano and Kemmelmeier, 2025). Their status depends on admiration rather than intimidation. Leadership and prestige frequently align as prosocial influence strategies, whereas dominance predicts reduced cooperation and interpersonal strain (Suessenbach et al., 2019).

Although dominance, prestige, and leadership differ in their behavioral style and interpersonal consequences (Henrich and Gil-White, 2001), they should not be treated as wholly unrelated motives. Suessenbach et al. (2019) conceptualized them as distinct yet moderately correlated dimensions of a broader social power motive. This means that individuals who strongly seek social influence may pursue that influence through multiple partially overlapping routes rather than through only one strategy. In competitive environments such as eSports, players may be motivated to combine coercive assertiveness, competence-based status, and influence over collective action. This interpretation is also consistent with person-centered evidence showing that some individuals exhibit profiles marked by simultaneously high dominance, prestige, and leadership striving, rather than specialization in only one route to status (Li et al., 2024b). Thus, even though leadership and prestige are more prosocial in their typical expression whereas dominance is more coercive and conflict-prone (Cheng et al., 2013; Suessenbach et al., 2019), all three motives may still be positively associated because they reflect related forms of status-seeking within the same competitive environment. Accordingly, we expect positive interrelations among the three motives in HoK.

H1a. In HoK, dominance motive will be positively associated with prestige motive.

H1b. In HoK, dominance motive will be positively associated with leadership motive.

H1c. In HoK, prestige motive will be positively associated with leadership motive.

### Social power motives and gender traits in gaming

Gender-linked traits (i.e., masculinity and femininity) represent independent dispositional dimensions rather than proxies for biological sex (Bem, 1974, 1981), making them appropriate for examining psychological alignment with power motives. Masculinity encompasses assertiveness, competitiveness, and instrumentality, and is culturally associated with dominance (Courtenay, 2000). Empirical findings support this connection: masculine traits predict higher dominance motivation (Malonda-Vidal et al., 2021). Femininity, defined by empathy and cooperativeness (Bem, 1974), is expected to relate negatively to dominance and more positively to prosocial or prestige-based influence patterns. Leadership research similarly shows that both agentic (masculine) and communal (feminine) traits shape leader effectiveness (Gannouni and Ramboarison-Lalao, 2019).

These patterns are further shaped by gaming’s cultural context. eSports environments remain masculinized (Salter and Blodgett, 2012; Taylor, 2003), and disparities in autonomy, play experience, and peer treatment diminish long-term female participation (Borkowski et al., 2024). Cultural expectations about gender may influence how power is expressed more than whether it is pursued. Consistent with this view, women *HoK* players show no deficit in achievement motivation relative to men (Li et al., 2024a), and women’s leadership-related profiles vary across both masculine-styled and feminine-styled forms rather than reflecting reduced power striving per se (Sumra, 2019). Gender-trait profiles, not biological sex, better explain variation in power strategies. Research on “alpha” women identifies both masculine- and feminine-styled variants; neither exhibits elevated social dominance orientation (Sumra, 2019). Women often combine assertiveness with communal traits, using prestige or leadership rather than pure dominance. Men likewise vary: high-dominance men may express aggression, whereas high-prestige men may display humility or patience.

Thus, masculinity is expected to correlate positively with dominance and leadership due to its assertive and agentic components (Sumra, 2019). Masculinity may also be positively associated with prestige, because prestige is not based only on communality but also on the public demonstration of competence, achievement, and socially recognized effectiveness, all of which may align with agentic self-concepts in competitive gaming environments (Cheng et al., 2013). By contrast, femininity should be negatively associated with dominance and may be positively associated with prestige and communal leadership insofar as these motives can also be expressed through empathy, cooperativeness, and prosocial influence (Gannouni and Ramboarison-Lalao, 2019; Bem, 1974).

H2a. In HoK, masculinity trait will be positively associated with dominance.

H2b. In HoK, femininity trait will be negatively associated with dominance.

H2c. In HoK, masculinity trait will be positively associated with prestige.

H2d. In HoK, femininity trait will be positively associated with prestige.

H2e. In HoK, masculinity trait will be positively associated with leadership.

H2f. In HoK, femininity trait will be positively associated with leadership.

### Social power motives and in-game aggression

Dominance, prestige, and leadership represent related but distinct orientations toward social influence. Dominance involves gaining influence through control, intimidation, and coercion; prestige involves gaining influence through respect, reputation, and perceived competence; and leadership involves guiding or coordinating others toward shared goals. These distinctions are important for understanding why social power motives may have different implications for in-game aggression.

Dominance has a direct conceptual and empirical link to aggression. Individuals high in dominance motive show a greater tendency toward antagonistic behavior (Johnson et al., 2012). Online disinhibition (Suler, 2004) amplifies this tendency, enabling dominance motivation to manifest through toxic behaviors such as flaming, trash talk, and cyberbullying. Empirical work confirms that power- and dominance-driven players are more likely to bully others in games (Kordyaka et al., 2023). Given HoK’s similarity to other competitive MOBAs, dominance should be associated with elevated in-game aggression.

Prestige and leadership motives show different implications. Prestige-motivated players depend on maintaining a positive reputation and open aggression threatens status. Prestige correlates with moral self-presentation and concern for status loss (Grubbs et al., 2019), reducing incentives for toxic behavior. Leadership motive aligns with prosociality and cohesion-focused conduct, consistent with findings that collaborative orientations predict prosocial behavior while competitive orientations predict aggressive behaviors (Kim and Ortiz, 2024). These motives therefore should be unrelated or negatively related to in-game aggression.

Contextual features of online play intensify these patterns. Anonymity weakens sanctions, facilitating hostile expression, particularly among dominance-oriented individuals (Kowert, 2020). Interviews with Chinese gamers show how anonymity encourages hostile alternate personas and escalatory cycles of aggression (Liu and Agur, 2023). Cycles of retaliation (a core feature of dominance-oriented interaction) are described in both qualitative work and recent studies of toxic escalation (Liu and Agur, 2023; Zsila et al., 2025), echoing dominance frameworks that treat aggression as necessary for control (Sidanius and Pratto, 2004). By contrast, prestige and leadership motives may discourage retaliation because aggression can damage reputation, respect, and group cohesion.

H3a. In HoK, dominance motive will be positively associated with in-game aggression.

H3b. In HoK, prestige motive will be negatively associated with in-game aggression.

H3c. In HoK, leadership motive will be negatively associated with in-game aggression.

### Gender traits and in-game aggression

Psychological evidence consistently links masculinity to higher aggression and femininity to lower aggression or nonviolent conflict resolution. Masculinity norms promote aggressive responding; femininity norms promote empathy and inhibition of aggression (Reidy et al., 2009). Empirical findings outside gaming support this: masculine orientation predicts verbal and behavioral aggression, while femininity predicts fewer hostile responses (Zeichner et al., 2009). These tendencies may generalize into gaming contexts.

Gaming’s sociocultural environment further shapes these patterns. Competitive gaming has long been male dominated (Pan, 2023), and aggression is often normalized as part of competitive identity. Women players experience disproportionate sexual harassment (Trudgett-Klose and McLinton, 2024). Hostile behaviors, including misogynistic and racist slurs, persist within some communities (Fox and Tang, 2016; Zsila et al., 2022), reinforcing aggressive expressions of masculinity as status signaling. Masculinity as a social identity within gaming can therefore amplify aggressive conduct, particularly when such behavior signals belonging in a masculinized competitive space. Femininity, by contrast, is associated with sensitivity to interpersonal harm and lower acceptance of toxic norms.

Accordingly, we anticipate that masculinity will correlate positively with in-game aggression, while femininity will correlate negatively, reflecting both dispositional tendencies and contextually reinforced behavioral norms.

H4a. In HoK, masculinity trait will be positively associated with in-game aggression.

H4b. In HoK, femininity trait will be negatively associated with in-game aggression.

## Method

### Wave 1

#### Procedures and participants

Data were collected using a self-report questionnaire administered through Credamo on September 25, 2025, following informed consent and institutional ethical approval. Participants had to have played Honor of Kings within the past 3 months to be eligible for Wave 1. We selected this game because it stands as the dominant MOBA in China. Its domestic daily-active-user count exceeded 139 million in October 2025 (CGTN, 2025). Participants were assured anonymity and confidentiality and received 2 RMB upon completion. A total of 295 participants provided valid responses (39 participants were excluded because of short response times). Regarding demographics, weekly playtime distribution showed that 3–6 h per week was the most common category (*n* = 98, 33.2%), followed by 7–10 h (*n* = 79, 26.8%) and 10 or more hours per week (*n* = 82, 27.8%). Only 36 participants (12.2%) reported 0–2 h of weekly play, indicating that most of the sample were active HoK players. Rank distribution spanned the full competitive ladder. This distribution indicates that the sample covered a broad range of player skill, from lower-ranked casual or less experienced players to highly skilled competitive players at the upper end of the ladder. The most frequently reported level was King and above (*n* = 75, 25.4%). This was followed by Diamond (*n* = 53, 18.0%), Platinum (*n* = 46, 15.6%), and Gold (*n* = 43, 14.6%). Mid-to-lower tiers were less prevalent, with Silver reported by 22 participants (7.5%) and Bronze by 12 participants (4.1%). Some participants also reported Master/Star Glory (*n* = 44, 14.9%), representing very high-tier players within the ranked system. Participants reported diverse preferred roles. Preferred in-game roles were distributed across all five primary lanes. Jungle was most common (*n* = 73, 24.7%), followed by Mid Lane (*n* = 70, 23.7%), Solo Lane (*n* = 60, 20.3%), Marksman/ADC (*n* = 54, 18.3%), and Support/Roamer (*n* = 38, 12.9%). The age distribution was concentrated among young adults (*M* = 28.88, *SD* = 6.56, *Maximum* = 56, *Minimum* = 18). The most frequently reported ages were 25 and 30 years (each *n* = 23, 7.8%), followed by 22 and 27 years (each *n* = 19, 6.4%). Ages 24, 28, 31, and 32 were also well represented (frequencies between 17 and 18). In total, 64.7% of participants were 30 years old or younger, and 82.7% were 34 years or younger, indicating a predominantly young adult sample. Only a small proportion of participants were aged 40 or above (5.7%), and very few were above 50 years. Gender was approximately balanced, with 155 males (52.5%) and 140 females (47.5%). Educational background ranged from high school to postgraduate study, with the majority (72.9%) reporting a completed or ongoing undergraduate degree, followed by those with postgraduate education and fewer with high-school-level qualifications. Together, the sample represents a broad range of active HoK players in terms of age, gender, rank, weekly playtime, and in-game role specialization.

#### Measures

##### Dominance, prestige, and leadership motives

Participants’ social power motives were measured using the adapted *Social Power Motives Scale* (Suessenbach et al., 2019), including 4 items for dominance, 4 items for prestige (at Wave 1, one item was removed due to low factor loading and inadequate reliability), and 4 items for leadership. Items were rated on a Likert-type scale (e.g., *1* = strongly disagree to *7* = strongly agree).

##### Gender identity traits

Gender identity trait was assessed using 12 items, six for feminine and six for masculine, from Bem (1981) Sex Role Inventory. Items assessed self-perceived alignment with gender-typical traits. Participants responded on a scale (e.g., *1* = strongly disagree to *7* = strongly agree).

##### In-game aggression

In-game aggression was measured using 9-item scales from the Buss–Perry Aggression Questionnaire (Bryant and Smith, 2001). Three domains were assessed: verbal aggression, anger, and hostility. Items were adapted to the gaming context where appropriate. Responses were provided on a Likert scale (e.g., 1 = strongly disagree to *7* = strongly agree). See Table 1 for descriptive statistics, reliability tests, and intercorrelations. The adapted items are provided in the Supplementary File A.

**Table 1:** Means, standard deviations, reliability, and intercorrelations of study variables (wave 1).

Variables	M	SD	McDonald’s *ω*	1	2	3	4	5	6
1. Dominance	5.07	1.12	0.77	1					
2. Prestige	5.82	0.81	0.70	0.540**	1				
3. Leadership	5.12	1.34	0.90	0.776**	0.476**	1			
4. Masculinity	5.34	0.94	0.85	0.730**	0.458**	0.790**	1		
5. Femininity	5.53	0.92	0.88	−0.013	0.171**	0.004	0.010	1	
6. In-game Aggression	4.02	1.20	0.88	0.096	0.165**	−0.056	−0.032	−0.133*	1

### Wave 2

Wave 1 revealed patterned links among power motives, gendered traits, and in-game aggression, but a single wave left open the question of whether the same association patterns would reappear over repeated measurement. Dominance, prestige, and leadership showed high intercorrelations at Wave 1. Wave 2 therefore reassessed the same participant pool to examine whether the correlational and regression patterns observed at Wave 1 would reappear across an interval.

#### Procedures and participants

Data were collected using the same self-report questionnaire administered through Credamo on November 7, 2025, following informed consent and institutional ethical approval. Participants had to have played Honor of Kings within the past 4 weeks to be eligible for Wave 2. Participants were assured anonymity and confidentiality and received 2 RMB upon completion. A total of 194 HoK players participated in the second wave of data collection (12 participants were excluded because of short response times). Participant linkage across waves was verified using platform-generated unique respondent IDs, ensuring that all Wave 2 cases represent the same individuals who completed Wave 1. Regarding demographics, ages ranged from 18 to 56 years. Age distribution was again concentrated among young adults (*M* = 30.14, *SD* = 7.21, *Maximum* = 56, *Minimum* = 18). The most frequently reported age was 30 years (*n* = 18, 9.3%), followed by 25 years (*n* = 15, 7.7%) and 32 years (*n* = 15, 7.7%). Overall, 56.7% of participants were 30 years old or younger, and 79.4% were aged 35 years or younger, closely mirroring the age profile observed in Study 1. Only a small number of participants were aged 40 or older, and very few were above 50 years, indicating a predominantly young adult sample consistent with active HoK players. Gender distribution at Wave 2 remained relatively balanced, though it was somewhat less even than at Wave 1, with 108 males (55.7%) and 86 females (44.3%). Regarding weekly gameplay hours, the largest group reported playing 7–10 h per week (*n* = 60, 30.9%), followed by players reporting 3–6 h (*n* = 59, 30.4%) and more than 10 h (*n* = 51, 26.3%). Only 24 participants (12.4%) reported playing 0–2 h per week. Thus, more than half of the sample (57.2%) played at least 7 h per week, reflecting strong engagement levels. Rank distributions covered the full competitive spectrum. The most frequently reported rank was King and above (*n* = 46, 23.7%), followed by Diamond (*n* = 45, 23.2%), with additional representation in Platinum (*n* = 25, 12.9%), Gold (*n* = 27, 13.9%), Star Glory (*n* = 25, 12.9%) Lower ranks (Bronze and Silver) collectively accounted for only 12.9%, indicating that the Time 2 sample skewed slightly toward higher-skilled players compared with Time 1. Preferred in-game roles were distributed across all five primary lanes. Mid Lane was most common (*n* = 54, 27.8%), followed by Jungle (*n* = 52, 26.8%), Marksman/ADC (*n* = 30, 15.5%), Solo Lane (*n* = 34, 17.5%), and Support/Roamer (*n* = 24, 12.4%).

#### Measures

Wave 2 employed the same set of measures used at Wave 1. Using identical instruments across waves served two purposes. First, it allowed the study to examine whether the same general pattern of associations would reappear over repeated measurement. Second, it provided a limited indication of short-interval stability for the measured constructs. The repeated-wave design is therefore used here to evaluate the recurrence of key association patterns rather than to claim independent replication from a separate sample. See Table 2 for descriptive statistics, reliability tests, and intercorrelations. All statistical analyses were conducted using IBM SPSS Statistics, Version 31.

**Table 2:** Means, standard deviations, reliability, and intercorrelations of study variables (Wave 2).

Variables	M	SD	McDonald’s ω	1	2	3	4	5	6
1. Dominance	4.93	1.26	0.81	1					
2. Prestige	5.49	0.97	0.76	0.551**	1				
3. Leadership	5.13	1.40	0.93	0.752**	0.290**	1			
4. Masculinity	5.34	0.94	0.86	0.715**	0.336**	0.799**	1		
5. Femininity	5.57	0.89	0.88	−0.081	−0.018	−0.033	−0.032	1	
6. In-game Aggression	3.86	1.22	0.88	0.186**	0.359**	−0.124	−0.008	−0.196**	1

## Results

### Wave 1

#### Interrelations among dominance, prestige, and leadership in eSports

Following Cohen (2013), correlation magnitudes are described using conventional qualitative labels (e.g., small, moderate, strong) for ease of interpretation. Pearson correlations indicated that the three dimensions of social power motivation were strongly interrelated (See Table 1). Dominance was positively correlated with prestige, *r* = 0.54, *p* < 0.001, and leadership, *r* = 0.78, *p* < 0.001. Prestige was also positively related to leadership, *r* = 0.48, *p* < 0.001. These coefficients demonstrate that within the online game environment, dominance, prestige, and leadership form a tightly interconnected cluster of social-power traits rather than independent motives. H1a, H1b, and H1c were supported.

#### Correlations between social power motives and gender traits

Dominance, prestige, and leadership were each positively associated with masculinity. Dominance correlated strongly with masculinity, *r* = 0.73, *p* < 0.001; prestige showed a moderate association, *r* = 0.46, *p* < 0.001; and leadership demonstrated a strong association, *r* = 0.79, *p* < 0.001. Feminine traits displayed a different pattern: only prestige showed a small positive correlation with femininity, *r* = 0.17, *p* = 0.003. Dominance (*r* = −0.01, *p* = 0.826) and leadership (*r* = 0.00, *p* = 0.948) showed no significant relation with femininity. Overall, social power motives were more strongly aligned with masculine than feminine trait orientations. H2a, H2c, H2d, and H2e were supported, but H2b and H2f were not supported.

#### Correlations between social power motives and in-game aggression

Because dominance and leadership were highly correlated, additional checks were conducted to evaluate coefficient interpretation in the aggression models. A hierarchical regression was conducted predicting in-game aggression from the three social power motives after controlling for rank, weekly playtime, and primary role. The inclusion of dominance, prestige, and leadership significantly improved model fit, Δ*R*^2^ = 0.076, and was significant overall, *F* (6, 288) = 6.32, *p* < 0.001. In the final model, dominance was positively associated with in-game aggression, *β* = 0.28, *p* = 0.003, and prestige also showed a positive association, *β* = 0.23, *p* < 0.001. Leadership yielded a negative coefficient, *β* = −0.23, *p* = 0.029. Because dominance and leadership were strongly correlated, we conducted additional single-predictor models as complementary exploratory analyses to clarify the interpretation of the multivariable coefficients. Leadership did not show an independent association with aggression when entered alone after the control variables (*p* = 0.948). The negative leadership coefficient emerged only when dominance was included in the model. Dominance showed an independent association with aggression when entered alone after the control variables (*β* = 0.19, *p* = 0.004). Given the strong correlation between leadership and dominance (*r* = 0.776), together with acceptable but nontrivial collinearity diagnostics in the full model (leadership: VIF = 3.771, tolerance = 0.265; dominance: VIF = 3.044, tolerance = 0.329), the leadership coefficient is best interpreted as a conditional residualized effect rather than a broad zero-order association. H3a was supported, H3b was not supported (effect in opposite direction), and H3c received support only in the multivariable model.

#### Correlations between gender traits and in-game aggression

Collinearity diagnostics were within acceptable ranges for the gender-trait model. A second hierarchical regression examined whether masculinity and femininity were associated with aggression after accounting for rank, weekly playtime, primary role, dominance, prestige, and leadership. Adding the two gender traits significantly improved the model, Δ*R*^2^ = 0.032, and was significant overall, *F* (8, 286) = 6.20, *p* < 0.001.

Femininity negatively predicted aggression, *β* = −0.17, *p* = 0.002, whereas masculinity was not a significant predictor, *β* = −0.10, *p* = 0.317. These results suggest that feminine trait orientation is linked to lower aggression in the online game environment, while masculinity does not show a reliable association after covariates are controlled. H4b was supported but H4a was not supported (Table 3).

**Table 3:** Correlations and standardized regression estimates among social power motives, gender traits, and in-game aggression in an online game environment (wave 1).

Predictor/variable pair	*β*	*t*	*p*	Tolerance	VIF
Regression predicting in-game aggression (social power motives)					
Dominance – Aggression	0.284	2.987	0.003	0.329	3.044
Prestige – Aggression	0.234	3.473	<0.001	0.659	1.518
Leadership – Aggression	−0.232	−2.190	0.029	0.265	3.771
Regression predicting in-game aggression (gender traits)					
Masculinity – Aggression	−0.094	−1.002	0.317	0.337	2.965
Femininity – Aggression	−0.172	−3.081	0.002	0.951	1.052

Regression values are standardized β coefficients from hierarchical models controlling for rank, weekly playtime, and role. *N* = 295. For the social power motive model, Δ*R*^2^ = 0.076, *F*_change_ (3, 288) = 8.24, *p* < 0.001. Leadership did not show an independent association with aggression when entered alone after the control variables (*p* = 0.948); its negative coefficient emerged in the multivariable model that included dominance and prestige. Collinearity diagnostics in the full social-power model were acceptable but indicated substantial overlap between leadership and dominance.

### Wave 2

#### Interrelations among dominance, prestige, and leadership in eSports

Pearson correlations again showed a strong, coherent pattern among the three social power motives at Wave 2 (See Table 2). Dominance was positively correlated with both prestige, *r* = 0.55, *p* < 0.001, and leadership, *r* = 0.75, *p* < 0.001. Prestige was also positively related to leadership, *r* = 0.29, *p* < 0.001. As in Wave 1, these results indicate that dominance, prestige, and leadership form a consistent cluster of social-power tendencies in the online game environment. H1a, H1b, and H1c were supported.

#### Correlations between social power motives and gender traits

The three social power motives were all positively associated with masculinity. Dominance correlated strongly with masculine traits, *r* = 0.72, *p* < 0.001; prestige showed a moderate positive relation, *r* = 0.34, *p* < 0.001; and leadership showed the strongest association, *r* = 0.80, *p* < 0.001. In contrast, none of the social power motives were significantly correlated with femininity (all *p* ≥ 0.05). This pattern resembled that of Wave 1, showing that social-power orientations align consistently with masculine, but not feminine trait orientations. H2a, H2c, and H2e were supported, whereas H2b, H2d, and H2f were not supported.

#### Correlations between social power motives and in-game aggression

The Wave 2 aggression model was examined in the same way because dominance and leadership again showed substantial overlap. A hierarchical regression examined whether dominance, prestige, and leadership predicted in-game aggression after controlling for weekly playtime, primary role, and rank. The baseline covariates did not explain aggression, *F* (3, 190) = 0.47, *p* = 0.703. Adding the three social power motives significantly improved model fit, Δ*R*^2^ = 0.252, and was significant overall, *F* (6, 187) = 10.90, *p* < 0.001. In the full model, all three motives were significant predictors: Dominance was positively associated with aggression, *β* = 0.40, *p* < 0.001. Prestige was positively associated with aggression, *β* = 0.28, *p* < 0.001. Leadership yielded a negative coefficient, *β* = −0.58, *p* < 0.001. Because dominance and leadership were strongly correlated, we conducted additional single-predictor models as complementary exploratory analyses to clarify the interpretation of the multivariable coefficients. When leadership was entered alone after the control variables, its coefficient was only marginal (*p* = 0.061). Dominance showed an independent association with aggression when entered alone after the control variables (*β* = 0.21, *p* = 0.005). As in Wave 1, the stronger negative coefficient emerged when dominance was included in the model. Given the strong correlation between leadership and dominance (*r* = 0.752), together with acceptable but nontrivial collinearity diagnostics in the full model (leadership: VIF = 3.758, tolerance = 0.266; dominance: VIF = 3.414, tolerance = 0.293), this Wave 2 pattern again suggests a conditional residualized effect rather than a simple zero-order protective association. Thus, higher dominance and prestige were associated with higher aggression, whereas the leadership coefficient should be interpreted cautiously. H3a was supported again, H3c was supported only in the multivariable model, but H3b was not supported (effect in opposite direction; Table 4).

**Table 4:** Standardized regression estimates among social power motives, gender traits, and in-game aggression (Wave 2).

Predictor/variable pair	*β*	*t*	*p*	Tolerance	VIF
Regression predicting in-game aggression (social power motives)					
Dominance – Aggression	0.404	3.540	<0.001	0.293	3.414
Prestige – Aggression	0.284	3.736	<0.001	0.657	1.521
Leadership – Aggression	−0.579	−4.839	<0.001	0.266	3.758
Regression predicting in-game aggression (gender traits)					
Masculinity – Aggression	0.025	0.235	0.814	0.329	3.038
Femininity – Aggression	−0.191	−3.056	0.003	0.976	1.024

Regression values are standardized *β* coefficients from hierarchical models controlling for rank, weekly playtime, and role. *N* = 194. For the social power motive model, *R*^2^_change_ = 0.252, *F*_change_ (3, 187) = 21.18, *p* < 0.001. Leadership was only marginal when entered alone after the control variables (*p* = 0.061); its stronger negative coefficient emerged in the multivariable model that included dominance and prestige. Collinearity diagnostics in the full social-power model were acceptable but indicated substantial overlap between leadership and dominance.

#### Correlations between gender traits and in-game aggression

A second hierarchical regression tested whether masculinity and femininity predicted aggression after the same covariates. Adding the two gender traits improved the model, Δ*R*^2^ = 0.036, and was significant overall, *F* (8, 185) = 9.67, *p* < 0.001. Femininity negatively predicted aggression, *β* = -.19, *p* = 0.003, whereas masculinity was not a significant predictor, *β* = 0.03, *p* = 0.814. These results suggest that feminine trait orientation is linked to lower aggression in the online game environment, while masculinity does not show a reliable association after covariates are controlled. H4b was supported but H4a was not supported.

## Discussion

### Brief summary

Across both waves, the findings showed a consistent pattern of support for most hypotheses. For H1, all three predicted interrelations among the social power motives (H1a–H1c) were supported at both waves. For H2, masculinity was positively associated with dominance, prestige, and leadership (supporting H2a, H2c, and H2e). Femininity showed a small positive association with prestige at Wave 1 only, offering partial support for H2d. However, femininity did not negatively predict dominance (H2b) and did not positively predict leadership (H2f), leaving both hypotheses unsupported. For H3, dominance was positively associated with aggression across both waves, whereas leadership showed negative coefficients only when the three motives were modeled simultaneously. Prestige predicted higher aggression, contrary to H3b. For H4, femininity consistently predicted lower aggression (supporting H4b), while masculinity did not significantly predict aggression after rank, weekly playtime, primary role, dominance, prestige, and leadership were controlled, providing no support for H4a. Overall, most hypotheses concerning social power motives were supported, while predictions involving femininity and prestige showed mixed or contrary patterns. More broadly, these results suggest that aggression in competitive online environments is meaningfully patterned by how players pursue status and by the gendered traits they bring to those interactions.

### Intercorrelations among social power motives

Our findings revealed that dominance, prestige, and leadership motives were strongly intercorrelated among HoK players. This aligns with prior work suggesting these facets share an overarching drive for social influence. Suessenbach et al. (2019), for example, validated dominance, prestige, and leadership as distinct yet moderately correlated dimensions of a unified power motive in the general population. In our gaming context, the correlations were even higher, indicating that players who seek one form of status tend to pursue others as well. Such high overlap may reflect a general power orientation in competitive gaming: individuals desirous of in-game influence often simultaneously crave dominance, admiration, and authority. This is consistent with the notion that status-seeking is a fundamental human motive (Li et al., 2024b). Competitive multiplayer games create a microcosm where the dual pathways to status identified in evolutionary theory: coercive dominance and earned prestige frequently intersect (Cheng et al., 2013). Indeed, person-centered research has found many individuals combine high levels of all three motives. For instance, Li et al. (2024b) identified an “ultra-dominance” profile characterized by simultaneously high dominance, prestige, and leadership striving. Our data similarly suggest that HoK players with a strong desire for power tend to score high on multiple status motives rather than specializing in only one approach.

Notwithstanding their overlap, these motives are not completely redundant. The moderate correlations reported by Suessenbach et al. (2019) and others indicate each motive retains some distinct emphasis (e.g., dominance on intimidation, prestige on earning respect). The persistently high intercorrelations across two survey waves in our study strengthen the interpretation that the relationship is trait-like and stable, not a one-time measurement artifact. If anything, the slightly lower prestige–leadership correlation at wave 2 suggests some fluctuation. However, the core pattern remained: those who enjoy taking charge (leadership) or gaining admiration (prestige) also often crave dominance. One explanation is that success in the HoK environment may require players to combine prestige and dominance: demonstrating skill to gain respect while asserting authority to climb rankings or lead teams. Over time, these motives may mutually reinforce one another. A prestige-oriented player may adopt some dominant behaviors to steer teammates, while a dominance-driven player might also seek prestige through high performance. This interplay echoes the idea that real-world leaders often integrate multiple power strategies (Li et al., 2024b). It also resonates with status characteristics theory, wherein respected (“prestigious”) individuals often accrue influence that enables directive (“dominant”) actions, and vice versa.

### Correlations between social power motives and gendered traits

Eagly (2013)’s structural perspective holds that gendered behavior stems from the distinct social roles assigned to men and women. Because women are typically positioned in communal roles that emphasize generosity, care, and nurturance, they are expected to display those traits. In contrast, men are placed in agentic roles that stress autonomy, task-focused behavior, and assertive dominance (Littlejohn and Foss, 2009). Our findings extend this pattern to the mobile gaming environment, social power motives aligned with masculine traits and were largely unrelated to feminine traits. Higher masculinity predicted stronger dominance, prestige, and leadership motives. High femininity showed minimal or inconsistent links to these motives, matching gender-role theory, which holds that those who see themselves as more masculine report greater desires to dominate or lead (Kent and Moss, 1994). This pattern mirrors prior offline and online findings: masculine norms promote power assertion, while feminine norms emphasize a non-competitive and caring stance (Reidy et al., 2009). Adolescents adhering to masculine stereotypes display more aggressive and dominant behavior, whereas femininity relates to lower aggression (Malonda-Vidal et al., 2021). Masculine traits also predict leadership identification among young adults, while feminine traits alone do not (Sumra, 2019). The current results extend this to gaming: players seeking in-game status tend to show agentic, “masculine” orientations regardless of biological sex.

The prestige motive showed a small and unstable positive correlation with feminine traits. Prestige, unlike dominance, can be earned through prosocial behavior, consistent with communal values. Some respected players may build status through supportive actions. Leadership in some cultures benefits from combining agentic and communal traits (Gannouni and Ramboarison-Lalao, 2019). In this study, however, leadership motives correlated almost exclusively with masculinity, suggesting that HoK players who want to lead view leadership in agentic terms. Competitive gaming culture rewards assertiveness rather than nurturing approaches.

Gender stereotypes and self-selection shape these patterns. Competitive gaming has been historically male-dominated and tied to hyper-masculine norms (eSports Research Network, 2020). Leadership experiences in eSports are shaped by gender (Piggott and Tjønndal, 2023). This environment strengthens the link between power motives and masculinity. Female gamers participate in a space coded as male, yet they can be as achievement oriented as male players, contradicting stereotypes of casual or social motives (Li et al., 2024a). Trait orientation, not sex, predicts power motives. Women high in masculinity pursue dominance and leadership similarly to men high in masculinity. Women who emerge as “alpha” leaders typically exhibit masculine qualities, with masculinity strongly predicting alpha status (Sumra, 2019).

Overall, masculinity fuels status striving in HoK, while femininity neither promotes nor strongly obstructs it. The competitive environment attracts players comfortable with assertive behavior. Those emphasizing empathy show less interest in power. Communal traits may still aid some leadership styles, but rank-oriented pursuit remains primarily agentic. Further work could examine whether communal players can lead effectively without adopting stereotypically masculine behaviors.

### Social power motives and in-game aggression

One of the most interesting findings was that the three social power motives differed in their associations with in-game aggression. As hypothesized, a strong dominance motive was associated with higher aggression during play. Dominance-oriented players reported more frequent hostile behaviors. This result is highly consistent with theoretical expectations and prior studies. Dominance motivation inherently involves the use of intimidation, force, or aggression to assert social rank (McClanahan et al., 2022). Our data are consistent with the interpretation that, in the context of online competitive gaming, dominance-striving players are more likely to engage in aggressive in-game behavior. Aggression and competition may “go hand in hand,” as Buss and Shackelford (1997) put it, functioning to increase one’s status or power in a hierarchy. In a ranked game like HoK, a dominance-driven player may be more prone to flame a teammate who fails as a display of superiority. Such behaviors, while corrosive to the social environment, may also be experienced as exerting control or inflicting humiliation.

The leadership finding requires more caution. Leadership motive did not show a meaningful association with aggression when entered alone at Wave 1 and was only marginal when entered alone at Wave 2. Its negative coefficient emerged primarily when dominance was included in the same model. Because leadership and dominance were strongly correlated at both waves, the appropriate interpretation is not that leadership directly reduces aggression, but that the component of leadership distinct from dominance was associated with lower aggression. This pattern is compatible with the view that some forms of influence striving in team games are more coordination-oriented and less coercive. However, the present results do not warrant the stronger claim that leadership straightforwardly suppresses aggression in eSports settings.

The most intriguing result was that the prestige motive also positively predicted aggression, contrary to our initial expectation. We had theorized that prestige-oriented individuals, concerned with earning respect, would avoid overt in-game aggression that could damage their reputation. Yet in both waves, higher prestige motive was associated with more self-reported in-game aggression. One reasonable reading is that, in highly evaluative competitive environments, prestige concerns do not necessarily operate in a uniformly prosocial manner. Players strongly oriented toward recognition and status may also report more antagonistic conduct when performance, rank, or reputation are salient. Research on status and aggression suggests that when reputation is at stake, even those who are not generally aggressive might aggress to defend or enhance their status (Bock and Brown, 2021). Our findings align with this notion of reputational aggression. The prestige motive entails sensitivity to others’ regard; thus, a prestige-oriented player may react strongly to being disrespected or losing rank. Fu (2019) indeed noted that competition for rank/status was a prime trigger of cyberbullying in games, with rank being “the number one reason” cited for aggressive or bullying behavior in game contexts (p. 4).

### Gendered traits as predictors of in-game aggression

When examining gender role traits, we found that femininity was a protective factor against toxic in-game aggression, while masculinity did not significantly predict aggression once other factors were controlled. In our regression models, players with higher feminine trait scores reported engaging in fewer aggressive behaviors, whereas players’ masculine trait levels showed no clear linear relationship with aggression frequency. This pattern aligns with a wealth of research linking femininity to gentleness and conflict avoidance. Individuals who consider themselves warm, compassionate, and sensitive to others (high femininity) are likely to restrain hostile impulses and seek non-aggressive solutions during interpersonal conflicts. Malonda-Vidal et al. (2021) provide supporting evidence: among adolescents, those with stronger feminine identification showed lower direct aggression, an effect mediated by better emotional regulation and empathetic self-efficacy. In other words, feminine traits contribute to skills and values (i.e., empathy) that help players keep their cool instead of flaming others. Our finding suggests these patterns may also be relevant in the gaming realm: a player high in femininity might be the one diffusing a tense situation rather than exacerbating it. Femininity’s negative link to aggression remained significant across two time points in our study, which suggests a relatively stable inverse association in this sample.

By contrast, we expected high masculinity to correspond to greater aggression, but after accounting for gameplay variables and motives, masculinity per se was not a significant unique predictor. This result is intriguing and somewhat counterintuitive considering traditional theories. One possible explanation is that masculinity’s influence on aggression is indirect or context dependent. Notably, masculinity was strongly tied to dominance motive in our sample, and dominance was a powerful predictor of aggression. It could be that masculinity is associated with aggression mainly through fostering dominance goals and a win-at-all-costs mindset. Once we consider those specific motives or the intensity of gameplay involvement (hours, rank), masculinity adds little further explanatory power. In simpler terms, being masculine may increase risk of aggression primarily to the extent that it is linked to becoming a highly competitive, dominance-seeking gamer, which our other analyses already capture. This interpretation aligns with Gan et al. (2024), who found that a gamer’s masculinity/femininity profile (i.e., social dominance) was more predictive of cyber-aggression than their biological sex, and that masculine traits alone did not always yield aggression unless paired with certain motivations.

The fact that femininity emerged as the only significant trait predictor of lower aggression highlights the vital role of empathy and interpersonal sensitivity in curbing aggressive behaviors. Feminine-identified players might refrain from lashing out because they are more attuned to others’ feelings or more troubled by causing harm. They may also internalize norms against aggression (e.g., “good team members should be supportive, not hostile”), which reduces their likelihood of acting out even under stress. In contrast, masculine-identified players, valuing assertiveness and dominance, might feel more license to express anger, yet in our data their aggression was highly variable. Some masculine gamers undoubtedly engage in toxic aggression (Horton et al., 2026). Importantly, the protective effect of femininity in our study was robust across sexes. Whether male or female, a player who describes themselves as tender, sympathetic, and gentle was less likely to report toxic behaviors. This underscores that it is the psychological traits, not gender itself, guiding aggression. A highly empathetic man may be far less aggressive online than a low-empathy (unfeminine) woman, despite gender stereotypes. Wright (2020)’s research showed that adolescents’ engagement in cyber aggression was better explained by their conformity to masculine or feminine traits than by their sex alone; for instance, youths with strong masculine-stereotyped traits perpetrated more direct online aggression, while those with more feminine traits engaged more in subtle or relational aggression and less in direct conflict.

### Limitations

Several limitations should be noted. First, the study relied entirely on self-report measures collected within the same survey context, which raises the possibility of common method bias. Although a Harman’s single-factor check was conducted as a preliminary diagnostic, this approach is limited and cannot rule out common method variance conclusively. More robust approaches would provide a stronger assessment and should be considered in future research. Second, the two waves came from the same participant pool rather than independent samples, so Wave 2 should be interpreted as repeated measurement rather than as an independent replication in the strong sense. Third, the play-history inclusion windows differed across waves: Wave 1 required play within the previous 3 months, whereas Wave 2 required play within the previous 4 weeks. This difference may have affected comparability. Fourth, although the present study used the second wave primarily for follow-up prediction and robustness checks, the two-wave design was not fully leveraged to examine temporal stability. Future research with longer intervals and additional waves should more directly assess rank-order stability, within-person change, and cross-lagged associations among gaming motivation, power motives, and in-game behavior. Fifth, the strong overlap between dominance and leadership complicates interpretation of their simultaneous regression coefficients. An additional limitation concerns the possibility of social desirability bias. Because the study relied on self-report measures of socially sensitive constructs, including in-game aggression, dominance-related motives, and masculinity/femininity traits, some participants may have responded in ways that presented themselves more favorably. This concern may be particularly relevant in the present cultural context, where reputational considerations and self-presentational norms may heighten sensitivity to such items. Accordingly, the observed associations may partly reflect self-presentation tendencies rather than only the underlying constructs of interest. Future research would benefit from incorporating behavioral indicators, peer reports, or other measures less vulnerable to social desirability effects.

## Conclusion

Across two survey waves of HoK players, dominance was the most consistent positive correlate of self-reported in-game aggression. Prestige also showed positive associations with aggression in the multivariable models, while femininity showed a consistent negative association. Leadership yielded negative coefficients only when modeled alongside dominance, indicating a conditional residualized effect rather than a broad zero-order protective relationship. Taken together, the findings suggest that aggression in this eSports context is most reliably associated with dominance-oriented status striving and that overlapping power motives require cautious interpretation when modeled together.

## Data Availability

The raw data supporting the conclusions of this article will be made available by the author, without undue reservation.
