# Supplementary material for: Social power motives, gendered traits, and aggression in eSports: evidence from two survey waves
Source: Front Psychol. 2026 May 13;17:1833661. doi: 10.3389/fpsyg.2026.1833661 (PMC13212060; doi:10.3389/fpsyg.2026.1833661)
Supplement: Supplementary file 1 [file Supplementary_file_1.DOCX]

**Common Method Bias Check (Wave 1)**

To examine the potential presence of common method bias, Harman’s single-factor test was performed using principal component analysis. The unrotated factor solution revealed four factors with eigenvalues greater than 1, explaining a cumulative 64.545% of the total variance. The first factor explained 28.918% of the variance, which is substantially below the threshold suggesting problematic common method variance. Therefore, the results indicate that common method bias is unlikely to threaten the validity of the findings.

**Common Method Bias Check (Wave 2)**

To assess common method bias, Harman’s single-factor test was conducted by entering all measurement items into an exploratory factor analysis. The results showed that four factors with eigenvalues greater than 1 were extracted, explaining 69.604% of the total variance. The first factor accounted for 30.844% of the variance, which did not exceed the critical threshold of 40% (or 50%). Therefore, common method bias was not a serious concern in this study.

**Measures**

***Dominance:***

I like to take the lead in decision-making within the game.

In order to win, I am willing to use aggressive tactics in the game.

If someone challenges my gameplay, I will do everything I can to defeat them to prove myself.

I want others in the game to follow my arrangements.

***Prestige:***

If my performance in the game is not recognized, I feel disappointed.

I feel very happy when I can show my skills or awareness to teammates.

I hope my gaming ability can be recognized by others.

Being respected and admired in the game is very important to me.

***Leadership:***

I like to take on the role of commander or pacesetter in the game.

I am not very interested in leading teammates in matches.

I feel very confident when directing the team.

I consider myself a competent in-game leader.

**Gender Identity:**

I consider myself to be warm.

I consider myself to be gentle.

I consider myself to be affectionate.

I consider myself to be sympathetic.

I am sensitive to other’s needs.

I consider myself tender.

I have leadership abilities.

I have a strong personality.

I often act as leader.

I consider myself dominant.

I defend my own beliefs.

I can make decisions easily.

***Aggression:***
In the game, I often have different opinions from my teammates.

If teammates criticize my gameplay, I can’t help but refute or argue back.

My teammates may feel that I like to argue in the game.

In the game, I can get emotionally agitated quickly, but I also calm down quickly.

Sometimes I suddenly lose my temper in the game, and afterwards realize it wasn’t necessary.

I find it difficult to control my temper in the game, especially when the situation is unfavorable.

I feel that I always encounter unfair matches in the game.

I feel that others always find it easier to win games than I do.

Sometimes I feel a strong sense of resentment or dissatisfaction with the game.
